# Simulation Studies of the Dynamics and the Connectivity Patterns of Hydrogen Bonds in Water from Ambient to Supercritical Conditions

**DOI:** 10.3390/molecules29235513

**Published:** 2024-11-21

**Authors:** Dorota Swiatla-Wojcik

**Affiliations:** Institute of Applied Radiation Chemistry, Lodz University of Technology, Zeromskiego 116, 90-924 Lodz, Poland; dorota.swiatla-wojcik@p.lodz.pl

**Keywords:** hydrogen bond, liquid water, supercritical water, MD simulation, flexible potentials, H-bond lifetime

## Abstract

Pressurized high-temperature water attracts attention as a promising medium for chemical synthesis, biomass processing or destruction of hazardous waste. Adjustment to the desired solvent properties requires knowledge on the behavior of populations of hydrogen-bonded molecules. In this work, the interconnection between the hydrogen bond (HB) dynamics and the structural rearrangements of HB networks have been studied by molecular dynamics simulation using the modified central force flexible potential and the HB definition controlling pair interaction energy, HB length and HB angle. Time autocorrelation functions for molecular pairs bonded continuously and intermittently and the corresponding mean lifetimes have been calculated for conditions ranging from ambient to supercritical. A significant reduction in the continuous and intermittent lifetimes has been found between (293 K, 0.1 MPa) and (373 K, 25 MPa) and attributed to the decreasing size of patches embedded in the continuous HB network. The loss of global HB connectivity at ca. (573 K, 10 MPa) and the investigated supercritical conditions do not noticeably affect the HB dynamics. Over the whole temperature range studied, the reciprocal intermittent lifetime follows the transition state theory dependence on temperature with the activation energy of 10.4 kJ/mol. Calculations of the lifetime of molecules that do not form hydrogen bonds indicate that at supercritical temperatures, the role of reactions involving an unbound H_2_O molecule as a reactant can be enhanced by lowering system density.

## 1. Introduction

The physical and chemical properties of water that are undoubtedly connected to the hydrogen bonding interactions have been systematically studied in a wide range of thermodynamic conditions using different experimental techniques, theoretical approaches and computer simulations [1,2,3,4]. Solvent properties such as the relative permittivity, viscosity, compressibility, heat capacity, electrical and heat conductivity and the ionic product strongly depend on temperature and pressure conditions, and can change abruptly near and above the critical point (T_c_ = 647.13 K, P_c_ = 22.055 MPa) [4]. Unlike ambient liquid, supercritical fluid is a poor solvent for ionic species and a good one for gases and hydrocarbons. The ability to modify the solvent properties through the temperature and pressure of the system makes water a highly tunable reaction medium with a broad range of applications in green sustainable chemical engineering [5,6].

The change in solvent properties is largely a consequence of the structural and dynamical hydrogen bond (HB) reorganization. Therefore, a characterization of these rearrangements at the molecular level is essential for understanding chemical processes in aqueous environments. Previous simulation works from this laboratory focused on the structural transformations taking place in the hydrogen-bond network (HBN) [7,8]. We studied the assembling of water molecules via hydrogen bonding using the modified central force flexible potential that includes short-range intermolecular interactions of the oxygen and hydrogen atoms and the anharmonic intramolecular part describing distortions from the equilibrium geometry [9]. This model potential better describes spatial hindrance resulting from the presence of hydrogen-bonding interactions. The extended-energetic definition of an HB [10] was assumed to decide whether a molecular pair was hydrogen-bonded. This definition simultaneously controls the interaction energy, the intermolecular O…H separation and the orientation of the H-donating partner. The population of hydrogen-bonded molecules was characterized by the probability distribution functions describing local hydrogen bonding, i.e., the number of HBs per molecule (n_HB_) as well as the number of hydrogen-bonded molecules in two connectivity patterns: nets or clusters and patches. A net was defined as a cluster comprising molecules having at least one H-bond to other members, whereas a patch as a supramolecular structure formed by continuously connected four-bonded molecules. We showed that these connectivity patterns are responsible for the structural inhomogeneity: patch-like, associated with the mean number n_HB_ > 2.0; and cluster-like, observed for n_HB_ < 1.9 [8]. Namely, at near-ambient temperatures, large patches are embedded into continuous gel-like HBNs. The size of patches steeply decreases with the increasing temperature and the decreasing density of water. The complete disappearance of patches at about 473 K is associated with breakage of the continuous HBN into a variety of hydrogen-bonded molecular clusters and the rapidly growing amount of H_2_O monomers, i.e., molecules not forming HBs. The cluster-like inhomogeneity is particularly pronounced at supercritical conditions, and strongly dependent on the conditions of temperature and pressure.

Over the years, the HB dynamics in water have been probed indirectly by a number of time-resolved spectroscopic techniques [1,11,12,13,14]. More direct quantitative insight into the process of HB breaking and HB making has been provided by molecular dynamic (MD) simulations [15,16,17,18,19,20,21]. Since experimental and computational studies have mainly concerned room-temperature or supercritical conditions, relatively little is known about the HB dynamics in between ambient and supercritical regions. The aim of this work is to reveal the interconnection between the dynamics of HBs and connectivity patterns resulting from structural rearrangements of HBNs. The same computational tools were used as previously [7,8] (see Section 4 for details). The investigated thermodynamic conditions are listed in Table 1. They refer to ambient water, pressurized liquid (373 K, 25 MPa; 473 K, 25 MPa; 623 K, 22.5 MPa), and supercritical water (653 K, 25 MPa; 673 K, 25 MPa).

Following the original concept of Rapaport [15], two types of time autocorrelation functions have been considered: continuous correlation functions for molecular pairs H-bonded without interruption over the time interval <0, t>, and intermittent ones for pairs not continuously bonded but restoring a broken H-bond within <0, t>. In addition to the above, time correlation functions of non-bonded molecules, i.e., not forming any H-bond to others within <0, t>, have been considered in this work. These non-bonded molecules are hereafter called monomers. The normalized time correlation functions represent the survival probability of H-bonded pairs (or monomers), and thus are related to the cumulative probability distribution functions of the lifetime, treated as a random variable [10]. The influence of the thermodynamic conditions on time correlation functions and the resulting mean values of the continuous and intermittent lifetimes, as well as of the monomer lifetime, are presented in Section 2. The relation between structural and dynamical rearrangements of HBNs is discussed in Section 3. Section 4 provides details of methodology, and Section 5 concluding remarks.

## 2. Results

### 2.1. Time Correlation Functions

The normalized time correlation function of hydrogen bonding has been defined as follows:

(1) $$H\left(t\right)=\frac{\sum_{i,j}{hb}_{ij}(t)\cdot {hb}_{ij}(0)}{\sum_{i,j}{hb}_{ij}(0)\cdot {hb}_{ij}(0)},$$

where hb_ij_(t)·hb_ij_(0) = 1 if a hydrogen bond between the i-th and j-th molecules existing at t = 0 is still present at time t > 0. Otherwise, hb_ij_(t)·hb_ij_(0) = 0. The summation runs over all molecular pairs bound at the instant t = 0. Although H(t) is the fraction of initially present hydrogen bonds which remains intact at a time t, Equation (1) also applies to track the time correlation of molecular pairs that restore temporarily broken H-bonds.

The normalized time correlation function of monomer persistence has been defined by Equation (2):

(2) $$M\left(t\right)=\frac{\sum_{i}{nb}_{i}(t)\cdot {nb}_{i}(0)}{\sum_{i}{nb}_{i}(0)\cdot {nb}_{i}(0)},$$

where nb_i_(t)·nb_i_(0) = 1 if the i-th non-bonded molecule existing at t = 0 remains non-bonded at time t > 0; otherwise, nb_i_(t)·nb_i_(0) = 0. The summation runs over all monomers present at the instant t = 0. In other words, M(t) shows what fraction of monomers present at t = 0 will survive until t > 0.

**Figure 1:**
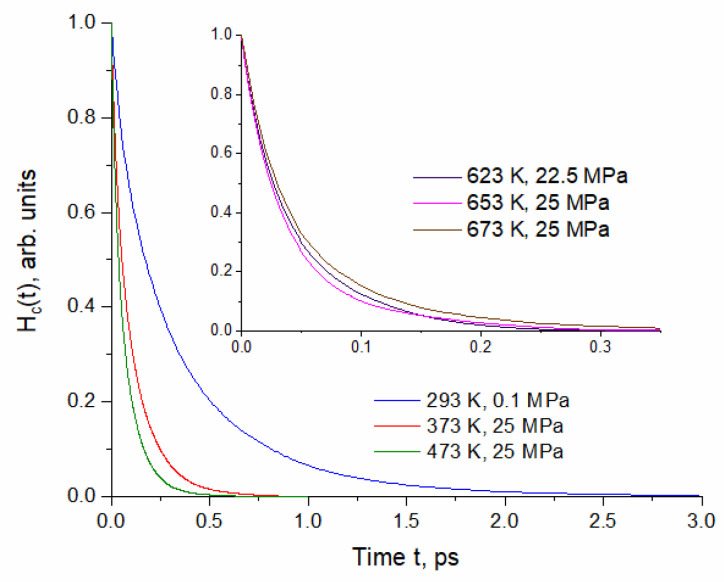
Selected normalized time correlation functions of continuous hydrogen bonding calculated for the thermodynamic conditions specified in Table 1.

The time correlation functions H(t) and M(t) have been calculated for the thermodynamic conditions listed in Table 1 by sampling equilibrium configurations as described in Section 4. Figure 1 shows the normalized time correlation functions of continuous hydrogen bonding H_c_(t).

The time correlation functions H_c_(t) show fast but non-single-exponential decay. Instead, all H_c_(t) functions are well reproduced by the two-exponential decay. The same feature was shown by H_c_(t) calculated assuming more linear HBs, i.e., HB angle α < 20° (see Section 4). This indicates a different behavior of H-bonded molecules, which is expected, given the previously demonstrated structural heterogeneity [8].

**Figure 2:**
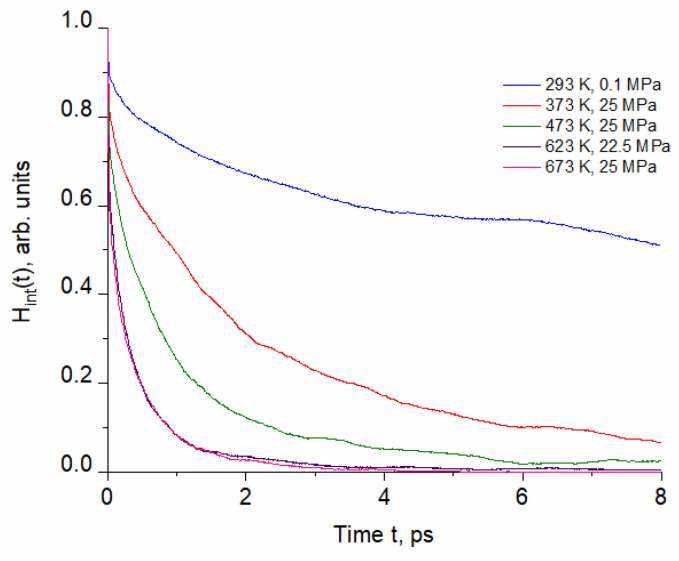
Selected normalized time correlation functions of intermittent hydrogen bonding calculated for the thermodynamic conditions specified in Table 1.

Figure 2 shows selected time correlation functions of intermittent hydrogen bonding H_int_(t).

Compared to H_c_(t), the behavior of H_int_(t) is different because H-bond making, breaking and reforming depend on rotational and diffusional motions. An interplay between self-diffusion and HB dynamics is responsible for non-exponential decay kinetics exhibited by H_int_(t) [16,22]. Unlike H_c_ function, H_int_(t) cannot be approximated by a two-exponential function. A significant change in H_int_(t) between (293 K, 0.1 MPa) and (473 K, 25 MPa) corresponds with the structural reorganization of the HBN [8] (see Section 1). At ambient conditions, H_int_(t) shows decay at short times, then inflexion, flattening and subsequent slow decay, indicating restoration of temporarily broken HBs. Such behavior corresponds with hindered translation and backward scattering of molecules in a rigid HBN stiffened by the presence of large patches [8]. Since this cage effect rapidly decreases with temperature of pressurized water, the characteristic flattening is much less pronounced. The time correlation functions H_c_(t) and H_int_(t) calculated for the subcritical and supercritical conditions are very similar, resulting in close values of the hydrogen bond lifetimes (see Table 2). 

**Figure 3:**
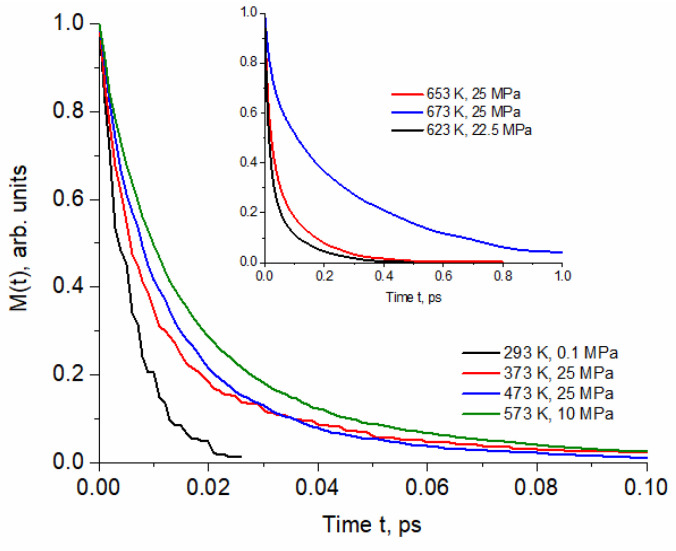
Selected normalized time correlation functions of monomer persistence calculated for the thermodynamic conditions specified in Table 1.

The normalized time correlation functions of monomer persistence M(t) are shown in Figure 3.

Similarly to the continuous correlation functions, M(t) can be well approximated by two-exponential decay dependence. The influence of thermodynamic conditions is particularly significant between ambient and pressurized water at (373 K, 25 MPa) and between (653 K, 25 MPa) and (673 K, 25 MPa) in the supercritical region. The former region coincides with significant reduction in size of patches and the more limited backscattering in molecular cages [8]. In this region, the significant change is also exhibited by the time correlation functions H_c_(t) and H_int_(t) (Figure 1 and Figure 2). In contrast to M(t), the H_c_(t) and H_int_(t) obtained for (653 K, 25 MPa) and (673 K, 25 MPa) are similar. Above the critical point, the water structure is very dependent on the density regulated by external pressure [23]. In particular, the low-density fluid is characterized by high structural inhomogeneity [24]. MD simulation of the (673 K, 25 MPa, 0.167 kg/L) system revealed the coexistence of a large number of monomers distributed in empty regions and clusters consisting of several H-bonded molecules [8]. The (673 K, 25 MPa) curve in Figure 3 shows that this structure favors long-term persistence of unbound molecules (see Table 2).

### 2.2. Lifetime of Hydrogen Bonds

The long time approximation $\underset{t\to \infty }{\lim}H(t)\approx exp(-t/\tau )$ often adopted to estimate the lifetime τ has the drawback that t →∞ is not accessible in simulation and, as shown above, H(t) is non-single-exponential at short times. This disadvantage can be avoided by using the method based on the basic statistical relations [10]. Given that H(t), by definition, represents the probability of survival of HB to time t, and considering the lifetime τ as a random variable that undergoes the normalized probability distribution function f(τ), the probability that τ will take a value greater than t can be expressed by the cumulative distribution function F(τ) as follows:

(3) $${H\left(t\right)=P}_{surv}\left(\tau >t\right)=1-F\left(\tau \right)=1-\int_{0}^{t}f\left(\tau \right)d\tau ,$$

Thus, $\frac{dH(t)}{dt}=-f\left(t\right)$.

The mean lifetime of HBs is expressed as follows:

(4) $$<\tau >=\int_{0}^{\infty }\tau f(\tau )d\tau$$

Integrating by parts and assuming H(∞) ≅ 0, one obtains:

(5) $$<\tau >=\int_{0}^{\infty }\tau \left[-\frac{dH(\tau )}{d\tau }\right]d\tau =\int_{0}^{\infty }H(\tau )d\tau ,$$

The statistical average of the mean values resulting from the integration of N = 5–8 correlation functions H_c_(t) and H_int_(t) has been calculated for the thermodynamic conditions listed in Table 1. The resulting values of continuous and intermittent lifetimes are presented in Table 2. The provided uncertainties correspond to 95% confidence intervals calculated from the Student t distribution for N − 1 degrees of freedom. 

The average lifetimes <τ_c_> and <τ_int_> decrease significantly between (293 K, 0.1 MPa) and (473 K, 25 MPa). Above 473 K, the decrease is smaller, and the values obtained for the supercritical conditions are the same within the statistical uncertainty. These results are consistent with the behavior of the time correlation functions H(t) discussed above.

The literature data reported from MD simulation are sensitive to the assumed model potentials, the HB definition, and the method of lifetime calculations [10,16,25]. For example the simple one-condition definitions are known to predict much longer lifetimes than the more restrictive two- or three-condition HB definitions. Moreover, as Table A1 shows, even the same type of definition leads to different lifetimes depending on the cut-off parameters assumed. It should be noted, however, that the values of <τ_c_> and <τ_int_> are within the range of the computational and experimental data reported for ambient and elevated temperatures [15,16,25,26] and for supercritical water [17,19,27].

**Figure 4:**
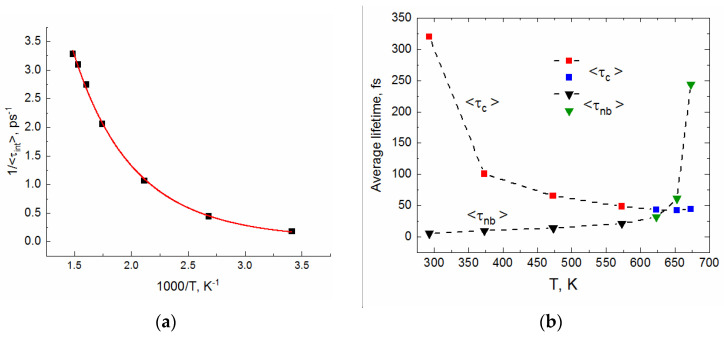
(**a**) The reciprocal intermittent lifetime <τ_int_> versus 1000/T (squares) and the non-linear fit to the TST dependence: $\frac{1}{<\tau_{int}>}=A^{\prime }T\;exp\left(-\frac{E^{\neq }}{RT}\right)$ [A′ = 0.032 ps^−1^; E^≠^ = 10.4 kJ/mol; Adj. R-square = 0.998] (red curve). (**b**) Temperature dependence of the average continuous lifetime of HBs <τ_c_> (squares) and the average lifetime of monomers <τ_nb_> (triangles). Lifetimes calculated for sub- and super-critical conditions are shown by blue squares and green triangles.

The results from Table 2 are depicted graphically in Figure 4. As can be seen, the lifetimes <τ_c_> and <τ_int_> steeply decrease between (293 K, 0,1 MPa) and (473 K, 25 MPa). At (473 K, 25 MPa), <τ_c_> and <τ_int_> are about five times shorter compared to their room-temperature values. In contrast to this, the HB dynamics at the investigated sub- and super-critical conditions are slightly dependent on temperature, suggesting an exponential dependence. Considering the HB dynamics in terms of reaction kinetics, the Arrhenius-like dependence of reciprocal lifetime might be expected. The Arrhenius dependence has not been found for 1/<τ_c_>, whereas the reciprocal <τ_int_> shows excellent fit to the transition state theory (TST) dependence: $\frac{1}{<\tau_{int}>}=A^{\prime }T\;exp\left(-\frac{E^{\neq }}{RT}\right)$ (Figure 4a).

The energy expense for the process of HB breaking and reforming is described by E^≠^. The fitted value of 10.4 kJ/mol is close to the activation energy of 10.8 kJ/mol assessed based on the light scattering experiments in the range 260 to 340 K [25,26,28].

### 2.3. Persistence of Monomers

Given that M(t) represents the probability of survival of non-bonded molecules, and considering the lifetime τ_nb_ as a random variable that undergoes the normalized probability distribution function f(τ_nb_), the same reasoning as in Section 2.1 leads to the following formula for the mean lifetime of monomers:

(6) $${<\tau }_{nb}>=\int_{0}^{\infty }\tau_{nb}f(\tau_{nb})d\tau \equiv \int_{0}^{\infty }M(t)dt$$

The average lifetimes <τ_nb_> obtained from integration of ca. 20 correlation functions M(t) are presented in Table 2. The uncertainty of each <τ_nb_> corresponds to the standard deviation. As illustrated in Figure 4b, the effect of temperature on the persistence of non-bonded molecules is opposite to that observed for <τ_c_>. Up to 573 K, <τ_nb_> gradually increases, following the linear dependence on temperature: <τ_nb_> = 0.0553·T − 10.991 (Adj. R-square = 0.975). On the other hand, the navy blue triangles show that the monomer persistence in sub- and super-critical water is very sensitive not only to temperature, but also to density. In particular, note the fourfold increase in <τ_nb_> between the (653 K; 0.451 kg/L) and (673 K; 0.167 kg/L) states.

## 3. Discussion

**Figure 5:**
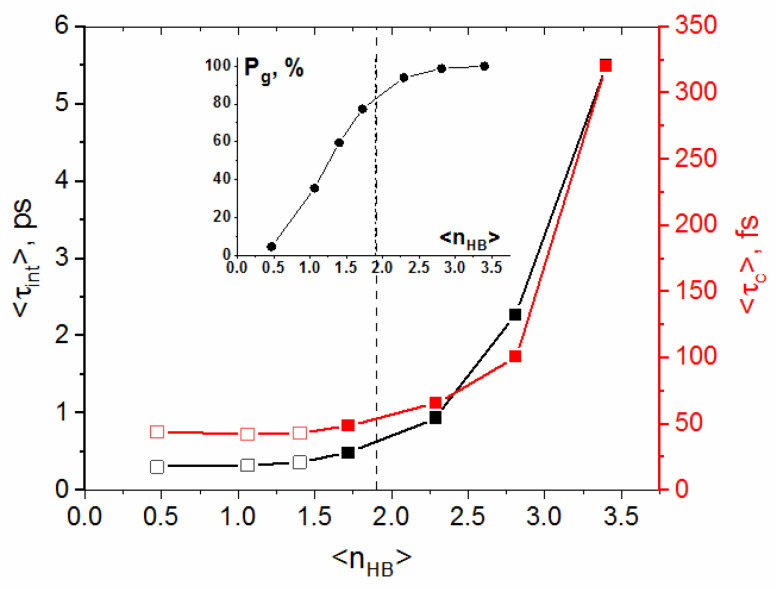
The calculated HB lifetimes versus the mean number of HBs per molecule (<n_HB_>): (black points and left axis)—intermittent; (red points and right scale)—continuous. Lifetimes calculated for sub- and super-critical conditions are shown by open squares. Inset: the dependence of a degree of connectivity (P_g_) defined as the total fraction of molecules engaged in the clusters of at least five hydrogen-bonded molecules [8]. The dashed line at <n_HB_> ~ 1.9 indicates breakage of the continuous HB network (right) into a variety of statistically independent clusters (left).

In Figure 5, the HB lifetimes from Table 2 are depicted as a function of the mean number of HBs per molecule <n_HB_>. The reduction in HB lifetimes occurs in the range where <n_HB_> decreases from its room-temperature value of 3.4 to 1.9. On the other hand, the relatively small change in <τ_int_> and <τ_c_> is seen for <n_HB_> lower than 1.9. The inset in Figure 5 shows the P_g_ parameter, introduced previously [7,8], to describe a degree of molecular connectivity via hydrogen bonds [7,8]. Namely, P_g_ was defined as the probability that a randomly chosen cluster of hydrogen-bonded molecules contains at least five molecules, irrespective of the number of H-bonds formed by each member. In other words, P_g_ shows the engagement of molecules in clusters composed of at least five H-bonded molecules. The inflection of the P_g_ (<n_HB_>) dependence at <n_HB_> ~ 1.9 coincides with breakage of the continuous gel-like HBN into a variety of statistically independent molecular clusters [8]. The loss of global connectivity at ca. 573 K (<n_HB_> ~ 1.8) is, however, preceded by a rapidly decreasing size of patches between (293 K, 0.1 MPa, <n_HB_> = 3.4) and (373 K, 25 MPa, <n_HB_> ~ 2.8) and the complete disappearance of these highly ordered supramolecular structures at ca. (473 K, 25 MPa, <n_HB_> ~ 2.3) [8]. Bond breaking in the HBN stiffened by the presence of large patches is more difficult, but its restoration is easier due to backward scattering. The sharp decrease in <τ_c_> and <τ_int_> is thus connected with the disappearance of large patches and, consequently, with the more flexible continuous HBN.

Between 473 K (<n_HB_> ~ 2.3) and 573 K (<n_HB_> ~ 1.8), a degree of connectivity in the pressurized liquid is still significant (P_g_ ~ 80–90%), but the influence of thermodynamic conditions on the HB lifetimes is not as much as in the presence of patches.

At the investigated sub- and super-critical states, the influence of thermodynamic conditions on <τ_int_> and <τ_c_> is small, even though the cluster-like inhomogeneity generates a steep linear decrease in P_g_ with decreasing <n_HB_> [8]. This result means that the dynamics of breaking and reforming HBs is slightly dependent on the degree connectivity in clusters and the number of HBs formed by individual molecules. It should be noted that if <n_HB_> falls below 1.9 (P_g_ < 80%), the static dielectric permittivity, the viscosity and the density of water are linearly dependent on the degree of connectivity P_g_ [7,8].

**Figure 6:**
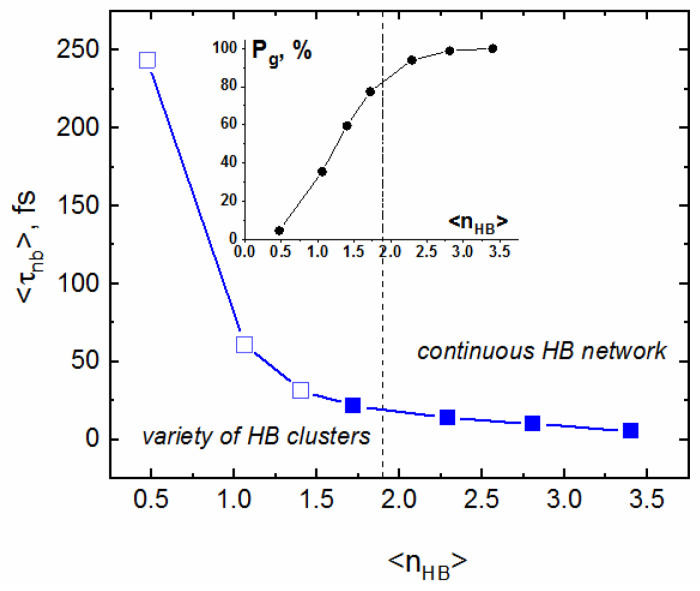
Persistence of non-bonded molecules (monomers) as a function of the mean number of HBs per molecule (<n_HB_>). Open squares correspond to sub- and super-critical conditions. Inset: the dependence of a degree of connectivity P_g_ on <n_HB_> as in Figure 5.

The dependence of persistence of non-bonded molecules (monomers) on P_g_ and <n_HB_> is opposite (Figure 6).

The lifetime of monomers in the continuous HBN is short and slightly increases with the decreasing P_g_. In the sub- and super-critical region, a degree of connectivity is very sensitive to water density (pressure). The cluster-like structural inhomogeneity is particularly pronounced in low-density supercritical water, where empty regions coexist with branched-chain clusters containing several H-bonded molecules [8,23,24]. At lower density (pressure) but at constant temperature, the number of monomers, as well as inter-monomer distance, increases, which makes intermolecular collisions less frequent, thus extending the lifetime of non-bonded molecules. As seen in Figure 6, at the supercritical conditions, <τ_nb_> is highly dependent on P_g_, and particularly long persistence of monomers is characteristic for the low-density system.

## 4. Materials and Methods

Simulation method. The NVE ensemble MD simulations were carried out for water under thermodynamic conditions specified in Table 1. The system was modeled by the periodically repeated cubic box containing 400 molecules described by the modified central force flexible model potential, including short-range intermolecular interactions of the oxygen and hydrogen atoms and the anharmonic intramolecular potential [9]. The equilibrium geometry and the partial charges −0.66e and +0.33e, located on the oxygen and hydrogen atoms, respectively, result in the dipole moment of 1.86 D. This model potential was used to ensure consistency with the previous simulation studies of structural transformations in HBNs [7,8]. The inclusion of flexibility, the short-range intermolecular interactions of hydrogen atoms, and the smaller partial charges offered by this model are advantageous for the description of hydrogen-bonded structures, particularly at elevated and high temperatures. The applicability of this potential to reproduce static and dynamic properties of water over the broad range of thermodynamic conditions has been discussed previously [7,8]. The size of the cubic box was calculated based on the experimental density of water at a given temperature and pressure. The initial configuration was obtained by random placement of water molecules in the cubic box and assuming equilibrium gas-phase geometry for each molecule. Initial velocities were sampled from the Boltzmann distribution. The equations of motion were integrated using the Verlet algorithm and assuming a simulation step of 0.1 fs. Long-range and short-range non-bonding interactions were treated by the Ewald summation method and the shifted-force method, respectively. Equilibration was performed by scaling of velocities monitoring temperature. To observe no trend in temperature, the equilibration stage required ca. 10^6^ time steps. Lengths of the production runs varied from 50 to 110 ps. Positions and velocities of the molecular sites were stored every 1 fs. The stability of the total energy was 10^−6^ < ΔE/E < 10^−5^. Temperature fluctuations characteristic for NVE simulation of the equilibrated system were within 10 K, with larger fluctuations observed for simulations of subcritical and supercritical states.

Hydrogen bond definition. The adopted HB definition controls (*i*) the pair interaction energy, E < −8 kJ/mol, (*ii*) the HB length, i.e., the distance between the hydrogen atom of the H-donor and the oxygen atom of the H-acceptor, R_O..H_ < 0.25 nm, and (*iii*) the HB angle α defined as the inclination of the OH bond of the H-donor to the line connecting the oxygen atoms, α < 30° [10]. The threshold values for E, R_O..H_, and α were proposed based on the pair energy distribution, the intermolecular part of the O-H radial distribution function and scattering experiments [7]. The same criterion was used to examine the persistence of monomers, i.e., molecules not forming H-bonds. Additionally, calculations of the HB lifetime were also performed assuming α < 20° (see Appendix A, Table A1).

Time correlation functions and lifetimes. Equation (1) was used to calculate normalized correlation functions for H-bonded pairs, whereas normalized correlation functions for non-bonded molecules were calculated from Equation (2). The calculations of continuous and intermittent hydrogen bonding correlations were performed using the time step Δt = 1 fs. The instant t = 0 was randomly sampled from the stored equilibrium configurations. Each correlation function represents an average of 100 independent samplings of the instant t = 0. Time integration of the survival probability (the normalized autocorrelation function), required to obtain the mean lifetime of HB (Equation (5)) or the mean lifetime of monomers (Equation (6)), was performed numerically using OriginPro 2019 software (OriginPro, version 2019 9.6.0.172, OriginLab Corporation, Northampton, MA, USA). The HB lifetimes given in Table 2 represent statistical averages of the mean values resulting from the integration of N = 5–8 correlation functions. The provided uncertainties correspond to 95% confidence intervals calculated from the Student t distribution for N − 1 degrees of freedom.

In the case of monomers, Δt = 1 fs was employed. The instant t = 0 was randomly sampled from the stored equilibrium configurations. The average lifetimes <τ_nb_> were obtained from integration of ca. 20 correlation functions M(t). The uncertainty of each <τ_nb_> corresponds to the standard deviation.

## 5. Conclusions

Information on the structure and dynamics of hydrogen bonds (HBs) is highly desired for understanding mechanisms of chemical reactions in aqueous media over the broad range of thermodynamic conditions. This work shows that the breaking and reforming of hydrogen bonds are strictly connected with the connectivity patterns found previously [7,8]. The significant reduction in lifetime observed between (293 K, 0.1 MPa) and (373 K, 25 MPa) is due to the decreasing size of patches (supramolecular structures formed by continuously connected four-bonded molecules) embedded in the continuous hydrogen-bond network. In turn, the loss of global connectivity does not have a major impact on the HB dynamics. At the sub- and super-critical conditions, the continuous and intermittent lifetimes are weakly dependent on temperature and density.

The novelty of this work is the calculation of the lifetime of monomers, i.e., molecules that do not form any hydrogen bonds. It has been shown that at supercritical temperatures, the stability of monomers can be significantly extended by lowering density (pressure), thereby increasing the role of chemical reactions involving an unbound H_2_O molecule as a reactant.

## Figures and Tables

**Table 1:** Thermodynamic parameters of the investigated states ^a^.

Temperature T, K	Density ρ, kg/L	Pressure, MPa
293 (288.3 ± 5.6)	0.997	0.1
373 (376.3 ± 6.8)	0.969	25
473 (474.2 ± 8.3)	0.881	25
573 (571.1 ± 9.8)	0.720	10 ^b^
623 (619.4 ± 10.0)	0.610	22.5
653 (649.5 ± 10.4)	0.451	25
673 (676.3 ± 10.2)	0.167	25

^a^ The value of density was assumed in NVE simulation; the mean temperature of simulation runs along with the standard deviation is given in parentheses. ^b^ Liquid-vapor coexistence curve.

**Table 2:** The average values of continuous (τ_c_) and intermittent (τ_int_) lifetimes of hydrogen bonds calculated for the thermodynamic conditions listed in Table 1. The last column presents the statistical average of mean lifetimes of non-bonded molecules (τ_nb_).

T, K	Pressure, MPa	<τ_c_>, fs	<τ_int_>, fs	<τ_nb_>, fs
293	0.1	320 ± 2	5500 ± 150	5.4 ± 1.7
373	25	100.7 ± 1.4	2269 ± 111	10.0 ± 2.7
473	25	65.7 ± 1.1	937 ± 17	13.9 ± 2.3
573	10	48.7 ± 2.4	486 ± 16	21.4 ± 2.1
623	22.5	43.3 ± 1.7	364 ± 23	31.8 ± 3.8
653	25	42.5 ± 1.8	323 ± 15	61.3 ± 6.9
673	25	44.1 ± 4.9	305 ± 13	244 ± 21

## Data Availability

Data is contained within the article.

## Appendix A

Table A1 presents HB lifetimes calculated using HB energy E < −8 kJ/mol, R_O..H_ < 0.25 nm, and HB angle α < 20° in the three-conditional definition of an HB.

**Table A1:** The continuous <τ_c_> and intermittent (τ_int_) lifetimes of hydrogen bonds calculated for the thermodynamic conditions listed in Table 1.

T, K	Pressure, MPa	<τ_c_>, fs	<τ_int_>, fs
573	10	23.9 ± 0.7	325 ± 14
623	22.5	22.1 ± 0.7	241 ± 9
653	25	21.2 ± 1.2	215 ± 11
673	25	24.5 ± 1.6	204 ± 10
